# NGMA-4. Creation of a MADR brain tumor single-cell atlas for examination of inter-/intratumor heterogeneity and the results of genetic perturbations in a diverse array of brain tumor subtypes

**DOI:** 10.1093/noajnl/vdab070.019

**Published:** 2021-07-05

**Authors:** David Rincon Fernandez Pacheco, Hannah Park, Katie Grausam, Joshua Breunig

**Affiliations:** Cedars-Sinai Medical Center, Los Angeles, CA, USA

## Abstract

We have recently established mosaic analysis by dual recombinase-mediated cassette exchange (MADR), which permits stable labeling of mutant cells expressing transgenic elements from precisely defined chromosomal loci. MADR provides a toolkit of elements for combinatorial labeling, inducible/reversible transgene manipulation, VCre recombinase expression, and genetic manipulation of human cells. Further, we have demonstrated the versatility of MADR by creating glioma models with mixed, reporter-identified zygosity or with “personalized” driver mutations from pediatric glioma. For example, introducing H3f3a (aka H3.3) mutation variants with MADR regulates the spatiotemporal profile of glioma, and single-cell RNA and ATAC sequencing analysis demonstrates a recapitulation of developmental hierarchy seen in K27M mutant human glioma. Moreover, we have generated novel models of H3.3 WT glioma, H3.3 G34R glioma, and supratentorial ependymoma using patient-derived oncofusion transgenes. These models display a high degree of phenotypic fidelity and we now compare these models on a single-cell level with our previous models, mouse single-cell RNA glioma datasets from other studies, and human tumor cell transcriptomes. Our multi-omics approach includes integration of ChIP-seq/Cut&Tag datasets, single-cell ATAC, and single-cell Cut&Tag datasets.

Moreover, we have engineered a novel methodology for inducible gain- and loss-of function perturbation studies in vivo. Using ETS transcription factors as a proof-of-principle, we overlay these genetic perturbations on the glioma atlas to examine the gene networks altered by precise molecular manipulations.

We hope that these combined approaches will enable researchers to discover disease mechanisms with increased resolution and test therapeutics in credentialed pre-clinical disease models.

